# A python’s embrace? Insurance and the global clinical trial

**DOI:** 10.1186/s12961-026-01449-6

**Published:** 2026-02-09

**Authors:** Janelle Winters

**Affiliations:** 1 Humanitarian and Conflict Response Institute, Ellen Wilkinson Building, School of Arts, Languages and Cultures, University of Manchester, Manchester, M15 6JA UK; 2 Centre for the History of Science, Technology and Medicine, Faculty of History, University of Oxford, Oxford, UK; 3 Pandemic Sciences Institute, Nuffield Department of Medicine, University of Oxford, Oxford, UK

**Keywords:** Clinical trial governance, Multi-country clinical trial barriers, Trial insurance policy, Research policy, Randomised controlled trial globalisation, History of norms/standards

## Abstract

**Background:**

Regulatory barriers present significant challenges to clinical trial approval during both “peacetime” and pandemics, particularly for multi-country clinical trials sponsored by academic institutions and low- and middle-income countries (LMICs). While such barriers have been depicted as a “python’s embrace”, analyses of trial approval efficiency and ethical frameworks have largely overlooked clinical trial insurance.

**Methods:**

I interrogate the evolution of clinical trial indemnification mechanisms, rationales, and operationalisation over the past fifty years through a structured literature review. I then consider the procedural barriers faced by academic institutions conducting multi-country clinical research during the COVID-19 pandemic using a case study of the University of Oxford’s “COPCOV” trial, which was led by the Mahidol Oxford Tropical Medicine Research Unit in Bangkok, Thailand. This includes thematic analysis of more than 65 semi-structured interviews with trial stakeholders and analysis of insurance documents from the Trial Master File and hundreds of stakeholder emails.

**Results:**

Supplementary reinsurance policies cost over £110,000 during the COPCOV trial, delayed trial approvals by up to nine months in some countries, and were largely justified by sponsors based on concerns about reputational damage. I argue that risk frameworks grounded in financial risk management and the commercial sector have expanded within academic institutions and, when coupled with an expansion of national requirements for “local paper” insurance policies, create serious barriers to initiating trial sites in many LMICs.

**Conclusions:**

Two potential reform pathways, which are grounded in procedural or systemic reforms and should be led by LMIC-based policymakers, could help to de-barrier clinical trial insurance procedures and ensure that evidence of efficacious (and affordable) countermeasures are available during future global health emergencies.

**Supplementary Information:**

The online version contains supplementary material available at 10.1186/s12961-026-01449-6.

## Background

Randomised-controlled trials (RCTs) provide the closest proxy to truth about whether a therapeutic is efficacious. This “magic” [35] relies on a delicate balance of risks. Because participants consent to assuming risks to their health or “wholeness” to contribute to society’s medical knowledge, bioethicists have long underscored the importance of research sponsors explaining and actively mitigating these risks [95]. Yet, clinical research by definition includes uncertainty and the risk of physical, psychological, or social harm can never be zero [99]. As part of a social contract of sorts, compensating participants harmed during a trial regardless of investigator negligence has been increasingly mandated globally over the past fifty years [31, 163]. Commercial trial insurance policies are the dominant mechanism for compensation. These policies are promoted through ethical language (e.g., respecting persons and promoting justice) and are designed as risk transfer mechanisms that also protect the financial and reputational assets of research sponsors [131].

Yet, strict mandates for documentation of insurance policies for each trial site raise their own risks. As a lawyer reflected in the 1960s, focusing on minimising risk at all costs would obstruct RCTs from being completed at all: “the purpose of clinical research is not necessarily to remain safe or to please lawyers or to satisfy bureaucrats or to avoid controversy or even to avoid lawsuits” [36]. RCTs rely on a delicate balance between providing adequate safeguards and keeping documentary approval thresholds manageable enough that investigators and populations from lower-resourced settings can participate.

This challenge is compounded during pandemics and other health emergencies, when the need for expediency in evidence production and uncertainty are both acute [110]. As miracle cures were promoted during the COVID-19 pandemic based on observational data [21], the WHO advocated for large, adequately powered trials with nimble (e.g., adaptive, platform, and pragmatic) designs that could provide evidence about repurposed drugs accessible in low- and middle-income countries (LMICs, [206]). However, echoing experiences during Ebola virus disease (EVD) in West Africa and the 2009 H1N1 influenza response [184], many multi-country COVID-19 clinical trials testing affordable countermeasures for COVID-19 – including the WHO’s Solidarity/Solidarity PLUS trial – missed peak caseloads in 2020–2021 due to approval challenges. Despite ethicists’ concerns about the negative consequences of excluding vulnerable populations from trials [23], fewer than two percent of COVID-19 RCTs included pregnant women [186, 203]. Virtually none included care home residents [82]. Many LMICs with high caseloads of COVID-19 were also heavily under-represented in trials [127].

**Fig. 1:**
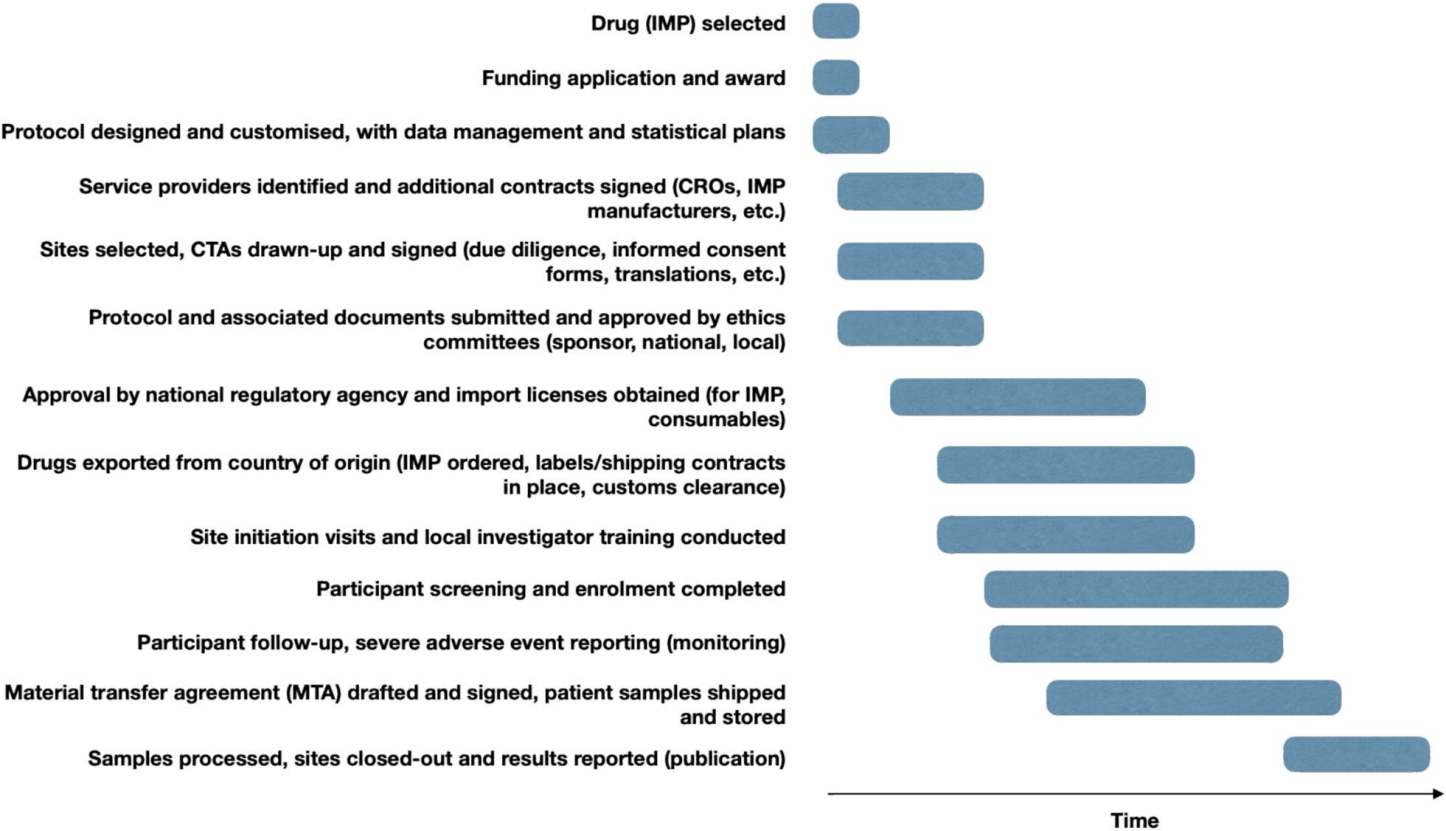
Steps in the clinical trial approval and implementation process that may present challenges. Trial insurance is typically listed in the protocol, included in clinical trial agreements (CTAs) with investigative sites, and considered as part of the CTA package by the ethics committee (and sometimes national medical regulatory authorities). IMPs are investigational medicinal products and CROs are contract research organisations. Shipments and storage of IMP or patient samples may be covered by insurance, but this would typically be under a separate policy

Researchers have identified barriers to conducting multi-centre trials at academic and non-profit institutions, particularly since the initiation of the International Council for Harmonisation of Technical Requirements for Pharmaceuticals for Human Use (ICH) in the late 1980s [62, 177]. They point to obstructive “document-based accountability” bureaucracies [162], “unintended consequences” of regulations [135], “over-regulation” [209], “regulatory capture” [1], and even regulators’ “censor’s hand” [180]. They also describe the role that these barriers play in excluding potential study sites in the Africa and Asia–Pacific regions [7, 148, 149]. Trial steps that commonly present barriers are shown in Fig. 1.

Yet, no known researchers have focused explicitly on RCT risk balancing through the lens of trial insurance. This article takes a step toward filling this gap, by exploring the globalisation of trial insurance requirements in the 1980s-2010s. It then uses a case study of a large, multi-country COVID-19 clinical trial’s insurance experiences to explore the barriers within this insurance system. The COPCOV trial, which was sponsored by the University of Oxford and led by the Mahidol Oxford Tropical Medicine Research Unit (MORU) in Bangkok, recruited 4652 participants in 2020–2022 from sites in eleven countries (Mali, Benin, Niger, Kenya, Cote d’Ivoire, Zambia, Nepal, Pakistan, Indonesia, the United Kingdom, and Thailand). COPCOV is an interesting trial from an insurance perspective because it was designed to test the antimalarial drug chloroquine/hydroxychloroquine as a prophylaxis against COVID-19. Chloroquine and hydroxychloroquine have a strong safety record, are affordable, and are widely available in low-resource settings globally [179]. COPCOV trial investigators approached 76 countries as prospective trial sites in early 2020 and submitted the trial protocol to 41 ethics committees or institutional research boards for approval. It took a median of 104 days (interquartile range 42) to receive an initial approval decision from each country, such that only two countries had activated and recruiting sites by the time vaccines were rolled out in early 2021. Insurance barriers contributed to these delays at many sites [207].

This article provides the first comprehensive analysis of an academic centre’s trial insurance experiences, including sponsor administration, financial costs, policy terms, and local investigator perspectives. My analysis draws inspiration from Whitney [199]’s framework that conceptualises trial risk management approaches as a “python’s embrace”. Whitney points to an over-emphasis of regulatory gatekeeper bodies on financial and legal risks over scientific and medical risks (in the case of insurance, the risk of excluding willing participants and of producing no timely evidence to guide therapeutics, particularly for vulnerable populations). The python’s embrace framework implies that ICH guidance is interpreted by national governments and risk guardians in ways that make it unfit for purpose for research outside of commercial settings. I also use Atuire’s framework of “de-barriering” research through critical trans-national discussions. De-barriering emphasises that fostering “pathways toward equal and level playing fields in global health will require examining the historical origins of existent barriers to participation” [194]. In the context of clinical trials, Atuire has argued that when actors in the Global North develop novel research ethics ideas and risk management frameworks – or “compulsions” beset by funders – these are often imposed on LMICs [14].

Ultimately, I argue that trial indemnification mechanisms are an under-studied contributor to a persistent “python’s embrace”. De-barriering the clinical research ecosystem is necessary to support multi-country clinical trials, particularly during health emergencies. This will require improved international guidance and transparency for trial insurance. It will also require deeper consideration of alternative mechanisms to commercial, sponsor-mediated insurance policies.

## Methods

The historical analysis is based on a literature review of published articles and reports on clinical trial insurance policy and practices since 1960 globally. The objective was to chronologically analyse major themes in the development of the current clinical trial ecosystem over time, and to characterise the actors and indemnification mechanisms that govern this ecosystem. Searches about clinical trial policy and practice were conducted in January 2025 in PubMed and Europe PMC (Supplement I lists the search protocol). Articles were included if they analysed policies or practices for clinical trial-specific insurance, indemnity, or compensation for injury in any geographic region and had an abstract translated into English. A literature rather than systematic approach was selected for the review because of the unfeasibility of rigorously searching all terms related to insurance over multiple decades across many geographies^1^. The challenges of a systematic approach are compounded by the changing use of these terms since the 1960s and the fact that relevant industry publications are not medically indexed. The review was supplemented by Google searches for grey literature, industry publications, and reflections on clinical trial insurance practices, using the search term “clinical trial insurance”. Additionally, it drew on primary sources about Good Clinical Practice (GCP) from the WHO Archives (Geneva), GSK Corporate Archives (London), and Novartis Corporate Archives (Basel).

**Fig. 2:**
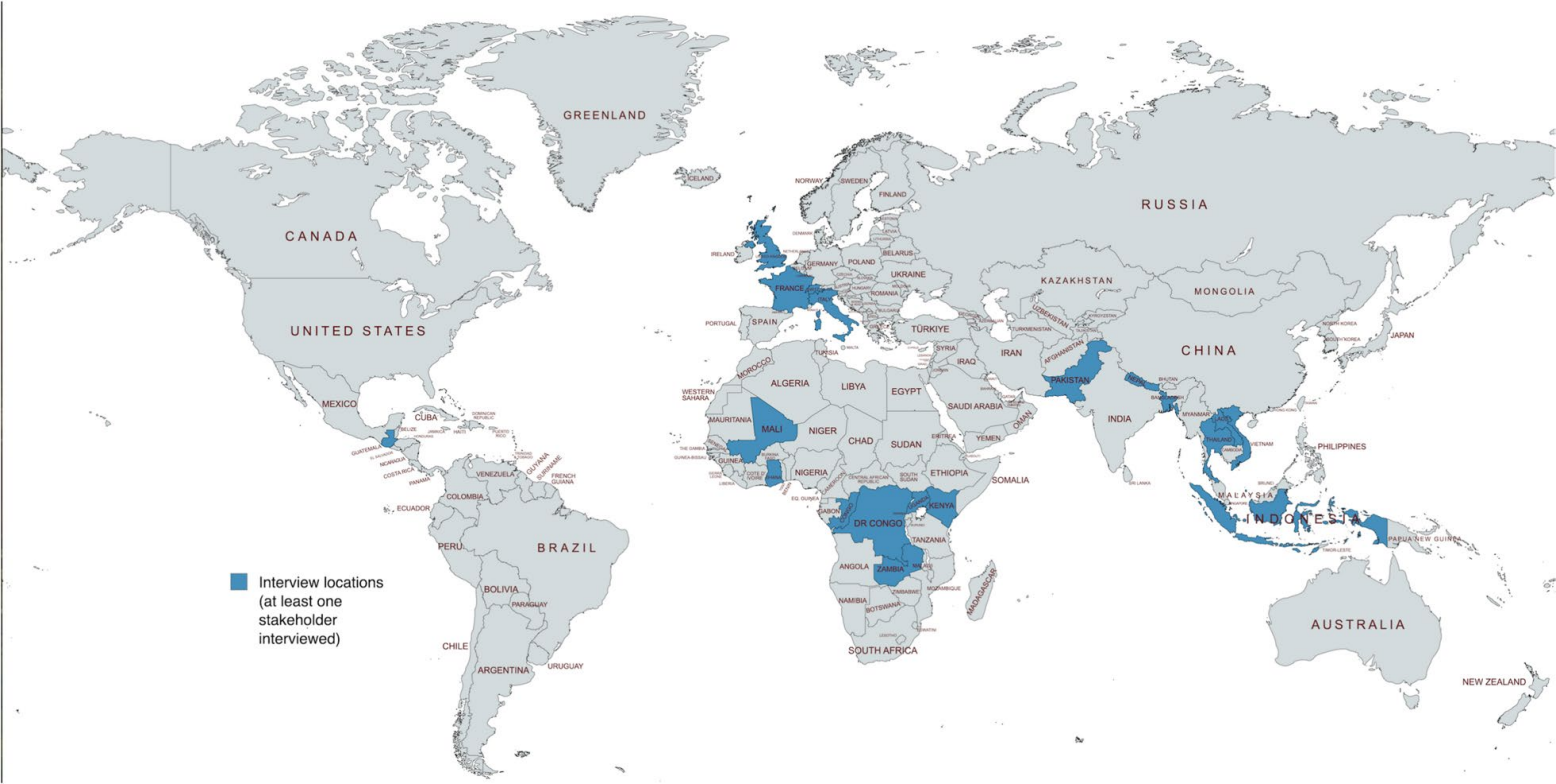
Stakeholder interview locations by country. COPCOV trial lead investigators at MORU contacted potential site investigators in 76 countries, of which 11 ultimately recruited participants. Stakeholders were interviewed in 18 countries, including those with prospective COPCOV sites (Ghana, Guatemala, Italy, Lao PDR, and Vietnam), active COPCOV sites (Democratic Republic of the Congo, Ethiopia, Indonesia, Kenya, Mali, Nepal, Niger, Pakistan, Thailand, United Kingdom, and Zambia) and stakeholders with broader knowledge of the clinical trial insurance landscape (e.g., WHO in Congo and Switzerland, Epicentre in France and Uganda)

The COPCOV case study is based on semi-structured interviews conducted with 65 global trial stakeholders from March 2022-May 2024, as well as informal discussions with four pharmaceutical and insurance company representatives from September 2023-December 2024. Interview details are provided in Supplement III. Interviews were conducted with permission from the University of Oxford’s Central Research Ethics Committee (CUREC R81146/RE001, for COPCOV interviews conducted in 2022–2023) and University of Manchester’s University Research Ethics Committee (UREC 2023–18356-32,399, for broader stakeholder interviews conducted in 2024). Investigators from countries that began any COPCOV regulatory or ethics submissions (regardless of whether these submissions were completed or approved) were invited to interview. Figure 2 shows the countries represented by at least one stakeholder interview. Interviews were aligned with the COREQ checklist, and based on questions about trial approval, site activation, recruitment, and closure activities. Interview guides included questions about insurance mechanisms in place during COPCOV and broader experiences with insurance for other clinical trials. Transcripts were analysed thematically, based on inductive coding in NVivo 15. Hundreds of COPCOV stakeholder emails and insurance documents from the Trial Master File were also analysed as part of the case study. These were shared with the author by trial investigators and study sites from January 2022-November 2023.

## Results

### Bioethics & early legal arguments for compensation

Legal and bioethics scholars began to discuss the issue of compensation for participants injured during clinical trials in the 1960s. While the Nuremberg Code (1947) underscored that the physician is always responsible for the subjects of a trial, it was silent about compensation for injuries [160, 145]. Most research and policy on the ethics of experimentation on human subjects, such as an influential article highlighting 22 cases of exploitative trials [19], focused on preventative and consensual aspects of trial ethics. This overlooked the fact that “despite all ethically prescribed precautions the subject may still suffer harm” [91]. Injured trial participants nearly universally relied on tort law to make financial claims. Tort law is an area of common law focused on civil wrongs and reliant on judicial precedent. Broadly, it “holds an actor whose conduct injured another – either through carelessness or, in exceptional situations, even in the absence of fault – liable for compensatory damages to the injured person” using the national court system [121].

Reliance on tort law was increasingly perceived to be morally and ethically questionable [30] because it required trial participants to surmount high upfront costs in a time-sensitive legal context [61] and could represent an unpredictable “litigation lottery” [95]. By the early 1970s, some legal and clinical experts presented formal cases for “no-fault” compensation funds for injured clinical research subjects, which would not require participants to prove investigators’ negligence [3, 173]. They argued that historical precedents, like insurance procured for the National Foundation for Infantile Paralysis’s Salk (polio) and rubella vaccine field studies and no-fault automobile insurance policies [91], demonstrated that “society has accepted the view that risk is reimbursable and those in hazardous occupations deserve extra pay” and the “cost of protection should therefore be considered a proper charge to the business of doing research” [20].

The Belmont Report (1979) [145] – and concerns that healthy volunteers suing for high damages under tort law would undermine the vaccine industry [83] – provided momentum for trial insurance discussions within and beyond the United States. However, like the Nuremberg Code and Declaration of Helsinki (1965), the Belmont Report has no concrete recommendations for compensation schemes (Table 1, [76]). As Meltzer [138] highlights, diverse interpretations of the Belmont bioethics principles have since fostered “incongruent” designs for trial liability mechanisms, which are not underpinned by a comprehensive ethical framework [159]. No-fault compensation is variously justified by “society owing a moral and ethical obligation to insured, based on principles of non-maleficence, beneficence, economic justifications, utilitarian justifications” [166].

**Table 1:** Global/multilateral guidelines for clinical trial compensation in the case of harm

Name	Recommendation/Guidance
World Medical Association’s Declaration of Helsinki – Ethical Principles for Medical Research Involving Human Participants (1964, 1975, 2013, 2024)	1964, 1975 – Did not address compensation issues 2013, 2024 – General principle 15 – “Appropriate compensation and treatment for participants who are harmed as a result of participating in research must be insured”
CIOMS/WHO’s International Ethical Guidelines for Health-Related Research Involving Humans (1993, 2016)	1993 – Compensation should be “equitable” 2016 – Guideline 14 stipulates that harm can be physical, psychological, or social and that participants should receive “free treatment and rehabilitation for such harms, as well as compensation for lost wages, as appropriate…regardless of fault”. This should be clearly listed in the protocol and informed consent and agreed upon before the trial commences, and each research ethics committee should “determine whether there is an adequate arrangement for treatment and compensation”
Council of Europe’s Convention of Human Rights and Biomedicine (1997)	Article 24 – Participants harmed are entitled to "fair compensation according to conditions/procedures prescribed by law” Additional protocol 35 – Compensation for harm should be “appropriate” (but there is no obligation to use insurance/indemnity to cover liability)
International Council for Harmonisation’s (ICH’s) Good Clinical Practice (1996, 2017, 2025)	Efficacy 6 (E6, Sect. 3.1.2), its revision (E6[R3], Sect. 3.14), and supplemental Efficacy 17 guidelines on multiregional trials – The research sponsor is responsible for providing ethics committees with information about compensation available to subjects and sponsors should provide insurance or indemnification for claims (excluding malpractice or negligence)
European Union (EU)’s Clinical Trial Regulation 536/2014 (entered into force 2014, fully replaced Clinical Trial Directive in 2022)	Article 76 – Requires that member states have a compensation system for any injuries caused in their territory (which can take the form of insurance, guarantees, or a “similar equivalent in nature and degree of risk”) Article 94 – Stipulates that member states should lay out rules for compensation implementation and penalties for infringement Article 95 – Places no limits on national or EU law in civil and criminal liabilities for sponsors/researchers (so contractual and tort law can apply for damages)

Based on [38, 48, 58, 76, 77, 95, 196, 100, 101, 102, 118, 129, 155] CIOMS is the Council for International Organizations of Medical Sciences

^a^Russia has a unique system; it charges a fixed price per participant for local insurance. Licenses are written on a tariff, based on personal injury policies (individualised, charged at set rate for participant, with only nationally licensed insurers allowed to issue them)

### Conceptualising liability in the ICH era

At least seven different indemnification mechanisms have been proposed for academically or publicly sponsored trials over the past fifty years, drawing on various bioethical principles (Table 2). In the 1980s, a push from transnational pharmaceutical actors for more integration across the three largest pharmaceutical markets, the United States, Japan, and Europe, resulted in the launch of ICH in 1988 and the release of its GCP guidelines in 1996 [2]. Advances in biomedical technologies and shifts in medical litigation increased the perceived risks of trials in these regions, particularly around contraceptive research [12], egg donation for stem cell research [189], and HIV/AIDS vaccine human trials [124].

**Table 2:** Proposed mechanisms for compensating participants injured during a trial

Mechanism	Description	Examples	Drawbacks/Challenges
Institutional compensation fund	A form of self-insurance, ideally based on no-fault principles	University of Washington’s compensation scheme (began as commercial insurance scheme via a special endorsement to the university’s comprehensive general liability insurance policy, and transitioned to a self-funded no-fault plan in 1979)	Requires the institution to have sufficient wealth reserves and awareness of national requirement(s) for compensation Ex: University of Washington trial participants must release themselves from tort action to proceed with a claim; medical expenses for injuries/economic costs are capped at about $10,000 (but free care at the institution)
Trans-institutional compensation fund	A form of pooled self-insurance, ideally based on no-fault principles, in which a group of research institutions band together (with or without government assistance) to establish a collective insurance pool	Laid out as an option in 1982 by the President’s Commission as a way of “harnessing flexibility” of self-insurance while distributing risk	Requires trust and strong, long-term relationship building between institutions, but could offer promise for less-resourced individual institutions to undertake clinical research
Nationwide compensation fund	A form of government or national organisation-mediated insurance, ideally based on no-fault principles	British Petroleum (BP)’s Deepwater Horizon and the September 11th Victim compensation funds appropriate funds from the federal government or private parties to be disbursed to a defined group of people injured, based on a specific set of severe adverse events	Requires a national bureaucratic structure and political will, and is easier to arrange for vaccines, retrospective injury-causing events, or trials with more predictable risks. May not incentivise sponsors/relevant actors to mitigate the risk of harm to research participants
Research or multilateral funding body-mediated	A form of research funder-provided no-fault indemnification, either through guarantees of directly assuming liability for non-negligent harm attributable to the trial sponsor or providing direct coverage of commercial insurance policy premiums	Proposals for the United States National Institute of Health or other major grant-funding bodies to provide direct costs for trial insurance policies or indemnify the trials themselves. In the United Kingdom, the National Cancer Research Institute has a no-fault clinical insurance	Momentum has been going in the opposite direction in countries like the United States and United Kingdom – the National Institutes of Health has long banned the use of its grants by local investigators to take out appropriate insurance as a direct cost even when insurance is required by local/national law, and the Wellcome Trust has recently prohibited sponsors from billing insurance as a direct cost
Commercially procured insurance (sponsor-mediated)	A form of insurance in which insurance policies are procured by a sponsor from a private/for-profit company, either on a per project basis or with broader coverage (e.g., per institution), ideally based on no-fault principles	Approach used in the early 1980s in Germany (with legal requirements to provide at least 500,000 DM in the case of death or permanent loss of ability to earn a living, paid by the sponsor of the trial based on a policy with a German insurance company) The 1982 President’s Commission’s consensus was that “blanket policies could be written and premiums set on the basis of the overall research mix and past claims experience of a given institution, although specific, high-risk protocols might require individualized underwriting examination”	Some commissioners and researchers argue this is the most flexible approach, puts positive pressure on institutions to quantify risk/deter riskier trials, and can operate voluntarily without national political will “Fine tuning” risk to bring down insurance premiums at academic institutions may stall research or be based on limited factual data, and as early as 1982 concerns were raised that additional costs/administrative burdens of insurance policies could stifle research, especially at less wealthy institutions
Specialty court for compensation	A form of no-fault compensation based on funds (often from taxes or other pooling mechanisms) allocated systematically to injured research participants through a specific independent body	The United States National Vaccine Injury Compensation Program, a no-fault programme for paediatric vaccines in place since the 1970s, is funded by a vaccine excise tax on each vaccine. It covers medical costs, attorney fees, lost wages, and pain and suffering claims, based on a vaccine injury table and a vaccine court	Requires national will and a bureaucratic structure, which may be hard to replicate across different types of clinical trials (particularly with nuanced determinants of injury causation) and countries. The vaccine court had mixed reviews, with a backlog of cases and relatively narrow list of injuries covered, and “immunises” vaccine manufacturers from liability (so may provide little incentive for sponsors to mitigate risks)
Direct insurance per participant	A form of no-fault compensation in which either universal health coverage (UHC) is applied or specific life and health insurance policies are procured for trial participants	Japan uses UHC/medical insurance to cover trial participation costs and the patient co-pay in the case of injury, and Russia and Ukraine mandate that sponsors purchase life and health insurance per trial participant at a fixed price to cover any injuries	Medical insurance policies may need to be clinical trial specific (or have a mechanism for preventing trial injury exclusions from standard health insurance, like for cancer trial participants). Finding insurance providers willing to switch to this model may be challenging; the 1982 President’s Commission’s survey of companies concluded that “the transaction costs and administrative burdens of writing coverage for individual participants in research were considered to pose insuperable problems in marketing such insurance”

Based on [3, 26, 34, 45, 61, 83, 95, 128, 138, 140, 161, 164]

While trial insurance policies began to proliferate in the “ICH market” countries during the 1990s, they were still far from universal. In the United Kingdom, for instance, the Association of the British Pharmaceutical Industry (APBI) issued its first guidelines for compensation for healthy volunteers injured in the course Phase I trials in 1970 and expanded these to Phase II-IV trials in 1983 [11]. However, a 1992 questionnaire of British research ethics committees indicated that only about sixty percent required no-fault compensation for *any* projects [90]. Clinicians also expressed concern that existing insurance policies were unfit for purpose. Barton et al. [18], for example, argued that some pharmaceutical companies sought to limit liability for injured trial participants by setting maximum payout amounts, vague limits on aggregate payouts, and requiring overly restrictive windows for claims. Within continental Europe, compensation requirements also often only applied to the for-profit sector [54].

A few highly publicised trial deaths and major lawsuits at American academic institutions in the 1990s and early 2000s tipped the scales and prompted more mandates for trial insurance [163]. As Mello et al. [136] describes, a heavily publicised gene therapy death and lawsuit at the University of Pennsylvania – the Jesse Gelsinger case – showcased how a previous era of “routine informed consent claims” expanded to include defective products, fraud, negligent conduct and monitoring, intentional infliction of emotional distress, breach of patient rights under local laws, and violation of national regulations claims. This also widened the net of who could be sued. Beyond research sponsors and investigators, institutional research board and data monitoring committee members became defendants in the Gelsinger case. The 2006 Northwick Park trial of humanised monoclonal antibodies in healthy volunteers further showcased the potential reputational damages of trials in the United Kingdom. The German company responsible for this trial, TeGenero, had a commercial insurance policy in place but attempted to deflect liability on the contract research organisation (CRO) responsible for the site, Parexel [208]. TeGenero had used APBI’s guidelines for its insurance provision, which are designed to align with damages that might be typically awarded in a British court and encourage arbitration schemes. There was public outcry when lawyers for the six injured men said clients – who had life-changing disabilities – were offered interim payments of £5,000 each, conditional on acceptance of the company’s arbitration mechanism, and when it was later revealed that the total trial liability insurance had a “wholly inaccurate” £2 million cap [49, 50].

With this widening net and the influence of ICH-GCP, academic research institutions increasingly included liability clauses in contracts, which themselves contracted an increasing number of commercial providers like CROs [142]. Trial insurance policy mandates and procurement also surged in the early 2000s in Europe. Between 2000 and 2012, 31 European counties mandated compensation for research-related injuries [163]. By 2004, when the European Clinical Trials Directive came into force, a broad array of legal obligations existed in member states. These ranged from legally enforceable direct claims against commercial insurers (e.g., Germany, Netherlands) to non-legally mandated but ethics committee-required policies (e.g., Belgium, United Kingdom) to voluntary or public institution-limited national no-fault schemes (e.g., Switzerland and Sweden, [47, 48, 119]).

### International indemnification globalisation

If tracking the various de facto and de jure insurance requirements in Europe presented a challenge for research sponsors in the mid-2000s, doing so globally became dizzying. Trial sites outside of the United States more than doubled from 1995 to 2005 [162, 163]. Beyond Europe, Australia, New Zealand, South Africa, and (to an extent) Singapore used APBI’s guidelines to guide industrial policies, as did Japan’s Pharmaceutical Industry Legal Affairs Association [120, 163, 189].

India’s clinical trial market grew especially quickly after 2005. This growth was incentivised by its rapid domestic and international generics pharmaceutical markets [5, 125]. By 2010 approximately one fourth of all clinical trials were conducted in the country, representing a $1.5 billion industry [183]. In India, the deaths of 49 children across six clinical trials conducted by an elite academic institution and two highly publicised deaths at a major CRO – as well as a 2011 government inquiry that revealed that several transnational pharmaceutical companies and major CROs had not paid compensation as required under law – led the Supreme Court to find that the government had failed to protect the rights of trial participants [87, 185]. International press also covered two lawsuits in Nigeria, one of the more rapidly growing clinical trial markets in Africa, against the pharmaceutical company Pfizer for deaths during a trial for bacterial meningitis [163].

Numerous countries and ethics committees in Africa followed suit in the 2000s and instated legal requirements for trial insurance. Yet, trial protocols continued to have widely variable descriptions (if any) of insurance details [52, 163, 190]. Such lack of standard language and understanding at many ethics committees of insurance fine-print reflects a stark lack of international proscriptive guidance or standards for clinical trial compensation (Table 1, [32]). Industry standards like APBI have been more influential than formal bioethics-grounded conventions or frameworks because they provide more tangible recommendations about the minimum terms for commercially procured insurance. Countries’ clinical research indemnification requirements have not been systematically tracked by the WHO or any non-industry global actor [31].

### Risk windows & protecting the trial marketplace

In many ways, clinical trial insurance policies in the corporate and transnational pharmaceutical setting arose independently from bioethics debates in the 1960s-1980s [159, 163]. Available archival records suggest that some of the first policies for trial indemnification were considered by companies in the early 1960s. Motivations were hardly altruistic. After meeting with an insurance company representative at Stuart and Lloyds in London in 1962, for instance, a Glaxo Laboratories (today GSK) legal department representative wrote that:

One can visualise circumstances in which some unfortunate consequences result in a patient taking part in a trial which are not the fault either of the manufacturer or the doctor. Technically, this would presumably come into the category of misadventure. In these circumstances, we might have to bear some legal and probably would have considerable moral responsibility. One of the most important points to emerge from the discussion was that so far as the law is concerned it is important that the individuals taking part in the trials should at least be told something of the nature of the investigation so that they are in law volunteers. Dr McDonald of Colindale told me that they have had this problem. Hitherto they have always brushed it aside and if the firm persists they have simply dropped the investigation. It does not seem, however, as though one could get away indefinitely with this cavalier attitude [86].

It is no coincidence that such concern over a “cavalier attitude” was contemporaneous with the early 1960s thalidomide scandal [126, 174]. Highly publicised product liability settlements exacerbated transnational pharmaceutical concerns about trial exposure windows [170, 172].

Tracing the evolution of insurance policies at transnational pharmaceutical companies is limited by non-disclosure agreements and secrecy within the “brave new world of privatized science” [1], also [168]. Available evidence suggests that trial insurance policies spread in tandem with the expansion of CROs and globalisation of trials – and the associated need of private actors to transfer and outsource risk to protect profits. Most early trial insurance coverage was part of broader insurance packages for product liability [27], but by the early 2000s the commercial insurance marketplace offered more targeted trial-specific policies. A 2003 industry-sponsored article situates trial insurance proliferation to the late 1990s, explaining that, “for years, clinical trials have been largely sheltered from the liability lawsuit crisis that has plagued other industries” but that recent increases in litigation meant that “companies must take aggressive steps to make sure the clinical trials they sponsor are above reproach and to guard against even the appearance of conflicts of interest” [10].

**Fig. 3:**
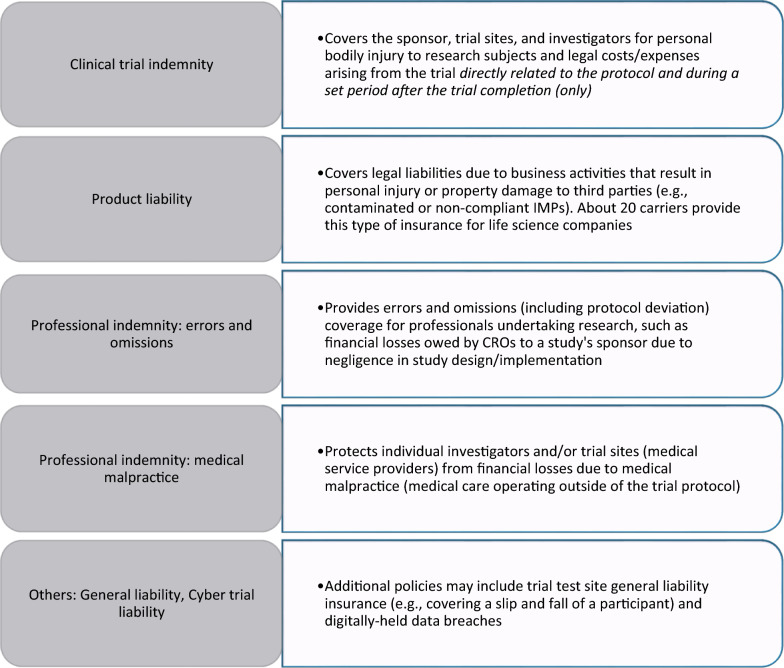
Categories of insurance policies applicable to clinical research

Figure 3 summarises the types of commercial policies now available to academic institutions, CROs, and pharmaceutical companies undertaking biomedical research and development. Broadly, medical malpractice insurance covers bodily injury to reach participants, professional liability covers this and financial harm to sponsors, and general liability covers “slip and fall” injuries (and is generally bundled with property insurance). Clinical trial-specific insurance combines features of medical malpractice and professional liability insurance [55]. In monetary terms, trial insurance policies represent a small component of both the wider international insurance and clinical trial marketplaces today. Some estimates place the global clinical trial marketplace at $80.6 billion in 2023 [85], with Phase III trials representing the largest revenue share within this market [147, 84]^2^ and CROs an “attractive asset class” for mergers and acquisitions [79, 165, 178]. In this context, it is productive to view trial insurance’s role as protecting liability exposure windows (a form of financial risk) and mitigating reputational damage for for-profit companies.

### The academic institution-mediated, commercial provider insurance system

**Fig. 4:**
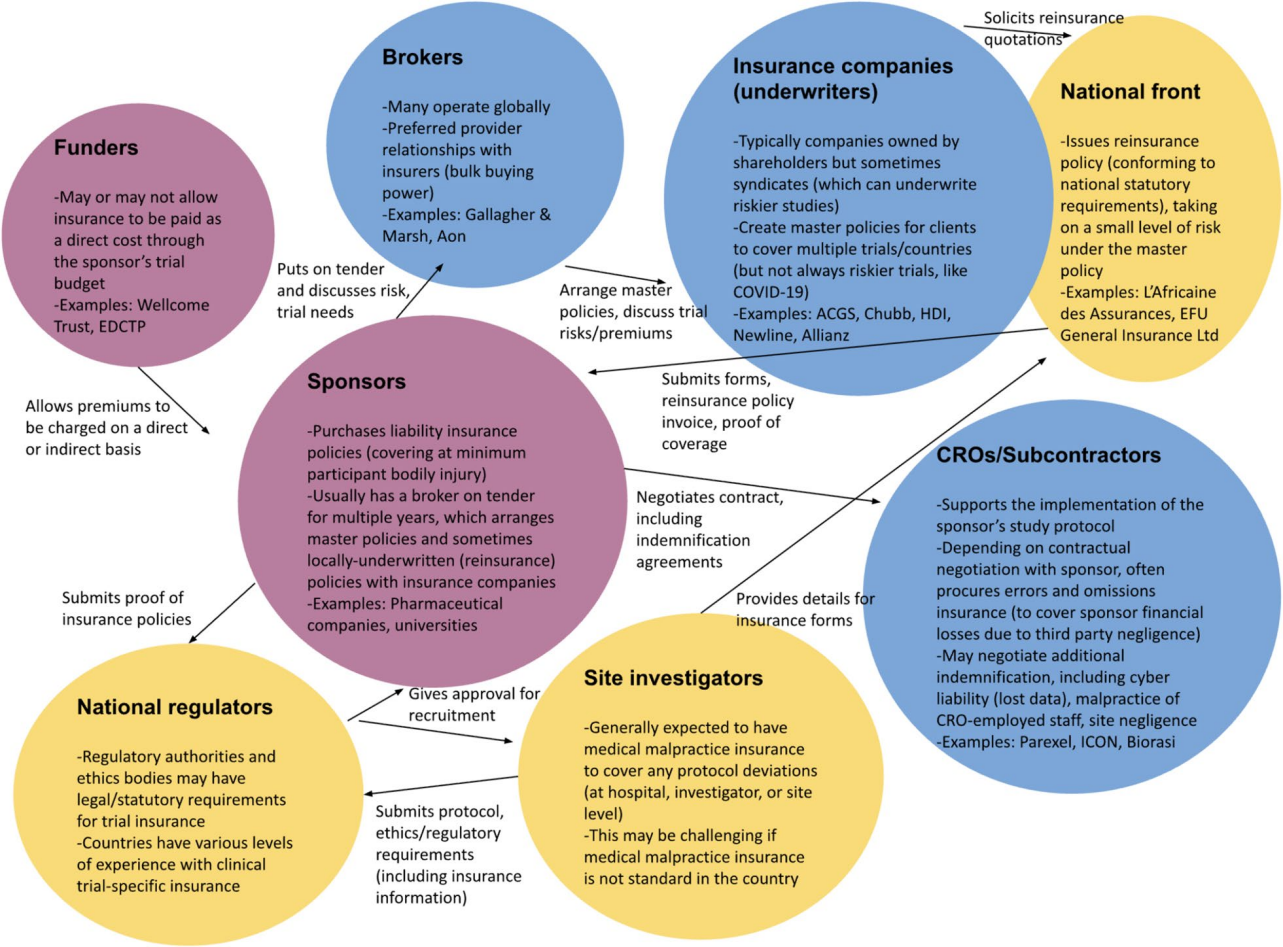
Major actors in the commercial insurance system. EDCTP is the European and Developing Countries Clinical Trials Partnership. Blue colour represents commercial actors, pink represents those financially responsible for procuring insurance (funding bodies and private or academic research institution sponsors), and yellow national-level stakeholders

As Fig. 4 shows, within the academic institution clinical trial insurance ecosystem, risk managers – rather than principal investigators or bioethicists – are the major mediators of policies. University research services teams generally typically place brokers on retainer to arrange a global master policy with an insurance company, which has its own underwriters. Some researchers from LMICs have been critical of this “insurance culture”. For instance, they have argued that, “we are putting these policies in place simply because they are an industry norm and commercial requirement in some countries” and that “in our country [Peru] there is no professional liability for our medically qualified staff and I do not think we should be introducing this as a means to maintaining the income of overseas firms” because it could “make trials even more expensive and out of reach for academic researchers in less wealthy countries”  [78].

Nebulous national requirements make academic sponsors increasingly dependent on the broker and master policy insurer’s know-how; as a university risk manager described, “having a broker, the broker networks as well, gives us access to more knowledge” (Interview 65). Brokers can be captive (representing only one insurance company) or independent and are typically paid by sales commissions from insurers [81]. Many trial brokers and insurance companies, in turn, use a proprietary third-party database, Axco, to monitor national regulatory changes within the insurance market for their clients (Interviews 36, 66). Axco charges for access to this database and had an annual revenue of $29 million in 2021 [16, 17]. Trials engaging populations deemed the highest risk by insurance company algorithms [8], particularly those including children with diseases like HIV/AIDS (which may require long insurance tail periods) and pregnant women, can be either uninsurable or require significant additional premiums [51, 137].

Based on the sponsor’s internal risk calculations; legal arrangements that the insurer has made with certain countries (national underwriting licenses); and national statutory requirements, sites taking part in a multi-country trial may be covered directly by the master policy or may require a local front to issue a policy, typically through “reinsurance”. One insurance expert described their transnational company as “fifty percent insurance, fifty percent reinsurance broker” (Interview 36). In the case of the insurance company Newline, for example, the parent company Lloyds Crystal approaches national insurance regulators on behalf of the syndicate to bargain for Lloyds insurance licenses. If the syndicate is successful in obtaining a license in a country, it can issue insurance directly (using its master policy).

When it does not have a license to write insurance in a particular country, the insurer must work through a local front to procure an admitted policy. In this scenario, the sponsor’s global master policy typically stands in “excess and difference in conditions” [16]. In other words, reinsurance policies are generally written at the minimum level required and backed by the international policy, with a small risk (often zero) assumed by the local insurance front. Risk managers have generally agreed that “if all countries accepted non-admitted cover, that would be so much easier…but then of course they want business written in their country because it brings money in”. This has created a situation in which “local insurers still try to add a bit of money on…charge them [the master policy insurer] $1,000 just to press a button” (Interview 66). The small profit margins for the local insurers can present an impediment to LMIC industry capacity building (Interview 35). An alternative would be for the sponsor or local sites to arrange an admitted insurance policy directly, without involvement of the provider of its master policy. However, this can be complicated by lack of experience with insurance fine-print; for a University of Oxford clinical trial in South Africa, for instance, an independent quote for a local policy was more expensive than the master policy holder’s quote and contained a relatively miniscule amount of indemnity (Interview 36).

### Insurance risk algorithms & fair payouts

Within this system, what is a “fair” or “appropriate” financial compensation for injury? The risk transfers underpinning trial insurance policies require complex quantitative negotiations about a range of complex ethically grounded questions. For instance, what “indemnity period” is acceptable – should policies cover the entire trial period (“life of trial”) [9] or can they be issued on an annual basis in some contexts, and why? How should the “indemnification threshold” or pay-out limit be determined, and should this differ in HICs and LMICs or in trials involving healthy volunteers? In what contexts should “tail periods” be mandatory, to cover claims after a trial ends? If private companies refuse to insure populations deemed the highest risk, what other mechanism could be used to include them [94]? Addressing these questions and developing algorithms to guide risk-based insurance premium costs and fair policy terms – as well as designing legislation in trial host countries to set minimum standards – requires data about past severe adverse events and payouts. For instance, what do courts consider the value of a human life, what is the likelihood that an insurance company will have to pay this, and how does this likelihood change based on scientific/medical attributes of trials?

There has never been publicly available data to rigorously address these questions, even within HICs [53, 138]. A 1976 United States federal task force found that “data on research-related injuries and on research subjects generally are extremely limited in terms of both the amount of information available and its generalizability” [164]. Thirty years later, the situation had not changed; Elliot [56] highlights that no oversight body tracks deaths or injuries of research subjects, that privately sponsored protocols and informed consent documents are confidential, and that the system of oversight “is so secretive it is difficult for watchdog organizations or journalists to investigate potential wrongdoing”. When revising the European Union’s Clinical Trial Directive in 2011, the European Commission further noted that there “are very limited figures on incidences of damage claims” in Europe [93]^3^.

No insurance companies have released their internal algorithms or data used to calculate the scientific/medical risk of trials and determine premiums. India is perhaps the only country that has a standardised, published algorithm (“quantum”) for compensation of injured trial participants and insurance policy costs. This algorithm was developed in the early 2010s based on a relatively simplistic quantification of risk, with compensation based on losses (so younger and healthier participants receive the highest payouts)^4^

$$Compensation (C)=\frac{Base Payout \left(B\right) x Age Factor \left(F\right) x Risk Factor (R)}{99.37}.$$

While some researchers have critiqued the quantum as overly simplistic in its accounting for risk, particularly for vulnerable populations like neonates, it promotes equity in the absence of statistical data about clinical trial severe adverse event incidence and payouts [133].

### The compounded challenges of multi-country trial insurance

Meeting insurance requirements can be particularly challenging for trials operating regionally and globally. In Europe, for instance, one regional trial described purchasing four separate policies to cover risks in EU member states in the early 2000s, with a total premium of nearly €60,000 [197]. The 2004 Clinical Trials Directive, designed to harmonise trials in the EU, contributed to *increases* in both fees for trial insurance and administrative costs [57, 171]. In the mid-2000s, for instance, a trial operating in eight European member states and Turkey required nearly two years of lead-time to meet harmonisation standards for the EU and spent €365,000 on insurance premiums [182]. Studies continue to highlight poor correlation between levels of trial risk and national or ethics committee trial insurance requirements and major differences in countries’ commercial policy terms [15, 26, 40, 93, 139, 167].

Insurance challenges are less documented for trials in the Global South. Minisman et al. [140] pointed specifically to the challenges of LMICs in procuring their own local malpractice insurance. Lai et al. [122], who provide the most comprehensive review of the drivers of start-up delays for global Phase III trials, found that “the most surprising area of potential start-up delay was clinical trial insurance…an area that is not widely discussed”. They highlight that varying country requirements and poor communication chains between clinical operations teams, CROs, insurance agents, and brokers can create a “great deal of potential for delay”. The COPCOV trial experienced such delays. Insurance was an early bottleneck for clinical trial protocol submission for national approval (see Fig. 1). Many countries had sequential review processes for ethics and national regulatory approval processes, during which insurance documentation was submitted. It took a median of 104 days (IQR 85 days, range 29–248 days) for a decision to be made by national authorities about the submitted COPCOV CTA for the initial protocol targeting health care workers. The protocol revision expanding to community recruitment after many health care workers were vaccinated was considered by national authorities for a median of 85 days (IQR 106 days, range 22–235 days, [207]). This resulted in COPCOV missing peak COVID-19 caseloads in most countries.

### Efficiency & adapting the system to emergency research needs

Efficiency is especially critical for clinical research in response to pandemics, which do not respect country boundaries. As summarised in Table 3, there have been some attempts to bypass the sponsor-mediated, private company-provided, country-based indemnification models during health emergencies. These have typically been limited to specific IMPs at the Phase IV trial or post-marketing stages. For instance, vaccine manufacturers asked the United States government to protect against claims during the 1976 swine influenza [146, 80, 29, 137, 144].

**Table 3:** Pathways used for expedited experimental drug and vaccine indemnification during major health emergencies. DPT is diphtheria-pertussis-tetanus. H1N1 vaccines are influenza vaccines

Mechanism	Examples	Challenges
Single country indemnifies drug/vaccine manufacturer	DPT vaccine in 1980s United States (vaccine excess law funds national compensation scheme)	Requires a complex legislative scheme to resolve injury claims fairly (operating outside of courts, but not covering negligence or supplier fraud) Nearly all LMICs and some HICs lack these national laws; governments usually useinterim/ad hoc arrangements during pandemics to assume liability (indemnity and ‘hold harmless’ agreements for manufacturers/suppliers)
United Nations uses its immunity from lawsuits to shield manufacturers	H1N1 influenza vaccines (some passed-through the WHO from manufacturers to countries, transferring certain legal liabilities to WHO)	The WHO or other United Nations agency must be the pass-through for any vaccines/IMP for this indemnity to apply The United Nations is shielded from lawsuits, but must take responsibility for any harm it causes through alternative means of settlement
International community establishes no-fault compensation fund	At least 19 jurisdictions had no-fault compensation funds during COVID-19	No major, global fund exists; the WHO proposed that the World Bank take the lead in indemnifying suppliers for an Ebola vaccine Any fund would have to have a fair liability cap, and countries would need to have excess policies to cover what a global fund would not

During the H1N1 response in 2008–2009, collective efforts for vaccine indemnification fell short. The WHO reduced the process of prequalifying vaccines for safety and efficacy from one to two years to as little as two days but it took an average of 100 days from when the donated vaccines were received for the liability arrangements to be formalized for African countries. In response to EVD in West Africa five years later, researchers argued that the “WHO’s traditional method of mitigating the legal risks through indemnification agreements with countries appears too slow to implement in urgent pandemic situations” [13]. This may reflect the lack of unallocated (core) funds at the WHO. Some policymakers therefore suggested a separate compensation system for Ebola experimental vaccine injuries based on no-fault principles, ideally overseen by a World Bank fund [117].

Suggestions that the World Bank or another multilateral actor provide indemnification for trials were not acted upon. When COVID-19 spread in 2020, stakeholders in the United Kingdom asked the national government whether they could indemnify national clinical trials because universities were taking on all liability for government-requested research that they sponsored. This did not proceed, leading a risk manager to reflect that, “if it can’t be done in house, I can’t see how it could be done worldwide…there’s just too many moving parts. I mean the World Bank possibly…but the liability would be huge” (Interview 66). The WHO’s Solidarity trial used a global liability mechanism that covered all countries and stakeholders. The COVAX Facility also created the “first and only vaccine injury compensation mechanism operating on an international scale” for COVID-19 vaccines. Both mechanisms were limited to specific IMPs or vaccines (and in the case of COVAX, did not include clinical trials, [39, 201, 202]).

Regional harmonisation bodies like the Pan-American Network for Drug Regulatory Harmonization and South-East Asia Regulatory Network do not address clinical trials [88, 175, 187]. The Africa region has a system for expediting clinical trial approvals during emergencies, the African Vaccine Regulatory Forum (AVAREF), but this process does not include insurance support [4].

Another option would be for trial funding bodies to provide indemnification mechanisms, particularly for research in response to health emergencies. However, momentum has largely been in the opposite direction. For instance, like the National Institutes of Health, the Wellcome Trust not only has no centralised indemnification mechanisms for trials it funds, but it has restricted the use of direct costs for insurance premiums since 2018. As a MORU operational leadership stakeholder described, “the only thing Wellcome rejected was the insurance… we tried to argue that it was site-specific because it was in the areas that we don’t usually work, but they said ‘no’, that it needed to come from non-core funds at either MORU or Oxford” (Interview 42, also Interviews 52 and 54). This can present challenges for local sites, who may have to unexpectedly fund thousands of pounds for a local insurance policy from their discretionary indirect funds (Interview 4). Mamotte et al. [128] and Cleaton-Jones [34] found similar challenges with National Institutes of Health-sponsored trials in Kenya, Malawi, South Africa, Tanzania, Zambia, and Zimbabwe [158], which required local investigators to find “innovative ways” to raise funds for procuring insurance policies.

### Case study of the COPCOV trial: is the system fit for purpose?

Within this insurance ecosystem, how did academic research institutions sponsoring large COVID-19 clinical trials make decisions about what form of risk they would assume and what compensation would be offered to study participants in the case of a severe injury? Some insurance companies would not underwrite COVID-19 trial insurance at all. Those that did often had to make internal arguments that stopping a major trial would present a reputational risk to the company (Interview 36). Trial sponsors generally had to pay extra pandemic premiums (Interview 66).

**Fig. 5:**
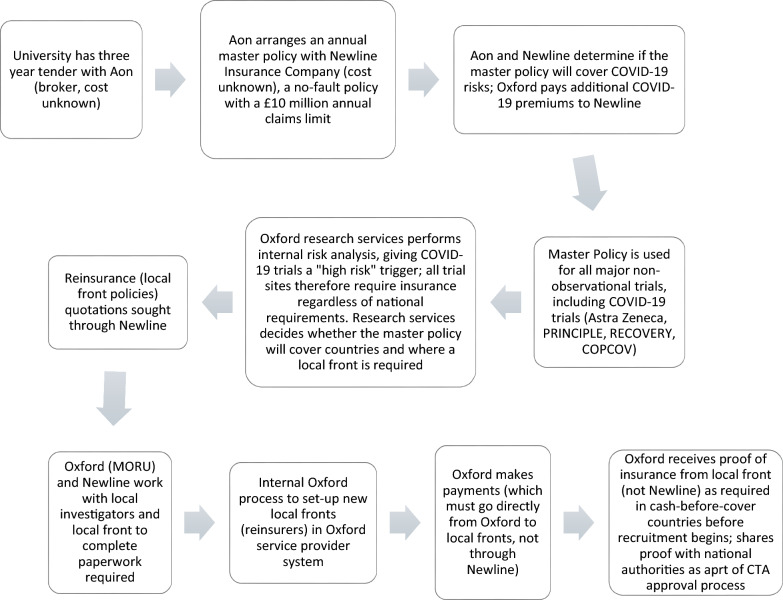
The University of Oxford’s process for insuring clinical trials during COVID-19

In the case of the COPCOV trial, the basic insurance governance was relatively transparent. As Fig. 5 summarises, the University of Oxford was under a three-year tender with the broker Aon at the start of the pandemic. Aon had prior experience providing insurance during pandemics [169] and had previously arranged a global master policy with the insurer Newline. Newline became one of the major providers of clinical trial insurance during the COVID-19 pandemic (in its words, “safeguarding solutions in risky times” by insuring at least 700 trials through three underwriting platforms, [151]). The Oxford master policy had a £10 million annual claims limit for no-fault liability, which covered participants enrolled in a study unless site investigators or CROs operated outside of the protocol. The same master policy was used for all major COVID-19 trials, including Oxford-Astra Zeneca, RECOVERY, PRINCIPLE, and COPCOV (Interview 66).

Decision making at the university about what clinical trial insurance would be required for prospective COPCOV trial sites was less transparent. Within the university, specific individuals became gatekeepers of the insurance procurement process. An individual in the university’s research services, who had recently joined the university from a corporate background in financial and risk assurances, decided that their predecessor had exposed the university to unacceptable risk by using the master policy to provide blanket cover in multi-country trials. Research services therefore put a risk trigger on all COVID-19 overseas trials [195]. Other triggers for study review included adaptive trial design, challenge studies, and any studies that involved gene therapy, children, or pregnant women (University of Oxford 2023). Overriding the risk trigger would require trial sponsors to escalate their case to the registrar, which could take months [67, 64, 108].

COPCOV investigators were therefore required to procure local insurance policies for all sites in countries that the master policy did not automatically cover [75, 154]. University research services decided that the broker and master policy companies should arrange this coverage, based on a perception that local sites lacked adequate insurance expertise to arrange appropriate policies and administrative capacity to handle potential claims (Interview 66). As shown in Table 4, which details Oxford research services’ compilation of national statutory requirements in countries with planned study sites as of March 2023, trial insurance cover was understood to be nationally mandated by just four of the 17 countries for which COPCOV procured insurance. However, only two countries, Lao PDR and Thailand, were non-admitted (i.e., would allow the University of Oxford’s master policy with Newline to cover a trial directly) and only the United Kingdom was directly listed on the master policy. Principal and site investigators were not given a choice about whether to procure insurance in the countries for which insurance was not legally mandatory, but for which the university would open itself to out-of-pocket liability in the event of a claim.

**Table 4:**
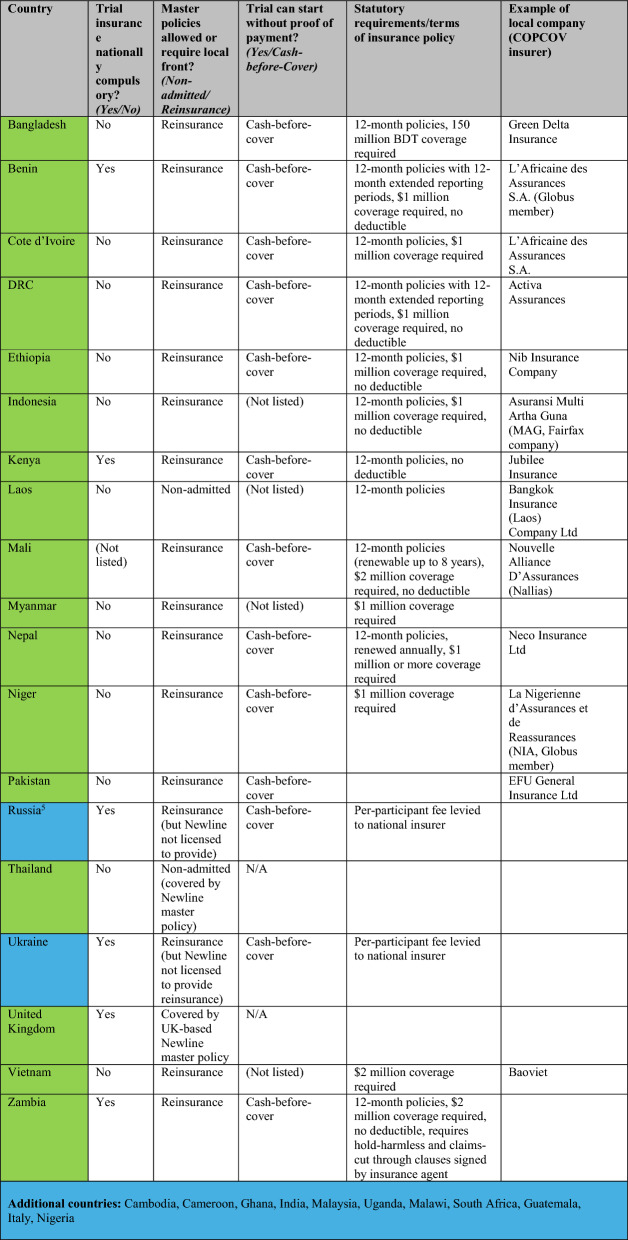
Tabulation of statutory requirements for clinical trial insurance during COPCOV

Green indicates a policy was put into force for COPCOV; blue that a quotation was received (but no policy was put into force). Countries fell into three categories: (1) non-admitted (covered directly by the master policy), (2) admitted but with no formal national requirement for insurance (i.e., insurance not mandatory but not covered by national policy, opening Oxford to financial exposure), and (3) admitted with a formal country requirement for insurance (i.e., Oxford required to procure local policy or reinsure via Newline). Table based on information compiled from University of Oxford research services’ "COPCOV overseas study status" Excel document, last updated in August 2022 and shared with JW in March 2023, and supplemented by additional information on local company policies from the COPCOV Trial Master File and stakeholder emails ^5^Russia has a unique system; it charges a fixed price per participant for local insurance. Licenses are written on a tariff, based on personal injury policies (individualised, charged at set rate for participant, with only nationally licensed insurers allowed to issue them).

In mid-March 2020, COPCOV principal investigators asked to move forward with quotations for reinsurance in all potential trial countries to minimise trial approval delays, even if this resulted in sunk costs [69, 154, 204]. From March 2020 through July 2021, the trial leadership team based at MORU in Bangkok; University of Oxford research services team; Aon; and Newline undertook a nearly continuous process of obtaining quotations and procuring reinsurance policies for these countries. The COPCOV trial ultimately spent approximately £110,000 purchasing local policies outside of its master policy (Supplement II)^5^. This is in addition to the global master policy cost (an undisclosed amount) and extra COVID-19 premiums levied by Newline (also undisclosed). Quotations were sought for at least 30 countries and reinsurance policies were set-up in 15 countries, of which 11 ultimately recruited participants. While insurance companies like Allianz have stated that “rates have been relatively stable…we have so far seen only moderate price increases on average, with higher price jumps for particularly exposed COVID-19 trials” [98], Newline’s risk calculations meant that extra costs for COVID-19 premiums were likely approximately half of the total annual, university-wide master policy cost (Interview 66, [150]). Oxford staff highlight that Newline showed good faith in its willingness to insure COVID-19 studies. Yet, the company’s algorithms for how they calculated risks guiding premiums were untransparent.

Procuring reinsurance for COPCOV was expensive monetarily, but the costliest expense was in delaying trial approval or site initiation. Recruitment at some sites extended until December 2021 and the results of the trial, which showed a small protective benefit for hydroxychloroquine against COVID-19, were not available until late 2022 and published in 2024 [179]. Some countries with COPCOV sites had months-long delays with site activation due to insurance bottlenecks. Table 5 summarises the insurance procurement barriers experienced during COPCOV, which were rooted in sponsor (University of Oxford), external party (broker, insurer, or funder), and national/local country requirements.

**Table 5:** Process barriers to insurance procurement for the COPCOV trial

Sponsor Level	Funder & Insurer Level	Local Level
Internal quotation requirements–	Sponsor as a “middleman”–	Cash-before-cover requirements–
Protocol amendments are required to add new countries before reinsurance quotations can be received. The process for seeking a reinsurance quotation can only be initiated 3 months or less from the intended date of recruitment	The trial sponsor (not trial sites) must be the named party on reinsurance policies, and it must pay for these policies directly. The insurance company with the master policy must solicit local reinsurance policy quotations	Most countries have cash-for-cover policy requirements; any delays in quotation processing and proof of payment lead to prolonged delays with site trial approval
Set-up of local fronts as suppliers–	Indirect communication channels–	Legal entity status requirements–
The local insurance company (reinsurance company) must be in the university’s system as a supplier before a payment can be made (the foreign insurer must send an invoice directly to the sponsor and then fill-in a supplier form in a specific format)	Local site investigators cannot communicate directly with local reinsurance companies (they must go through the sponsor, and the sponsor must go through the master policy holder, Newline)	Local sites or collaborating institutions must be registered as a legal entity for reinsurance premium to be paid; sites with limited clinical trial history who do not have this status must find a registered collaborator or they cannot be insured
International payments–	Funder restrictions–	Banking requirements–
Payment must be made from the local front to the trial sponsor directly (not through the local site, the insurance company, or the broker), using a purchase order. International payment must be made according to standard timelines (once weekly). Only the service provider authorisation team can see the status of payment	Some funders (e.g., Wellcome Trust) will not cover trial insurance as a direct cost; this requires local sites to use their discretionary overhead funds to cover reinsurance (and the sponsor must invoice the sites for these funds, since it must pay the reinsurance company directly)	Countries that hold capital at central banks or have strict currency requirements require significant negotiation about supplier forms and how to charge fees

Based on data from [43, 63, 69, 70, 73, 111, 114, 154, 191, 192, 205]; Interviews 48, 59, 66

These barriers were compounded by limited communication channels between local sites and insurers; a site investigator described the university as being forced to be a “middleman” between the master policy insurer and local fronts, with trial sites operating on the periphery (Interview 59). None of the administrative processes were expedited in response to the health emergency. Collectively, the barriers gave investigators narrow options to be geographically nimble during the pandemic or to select sites as the viral burden changed worldwide. This reinforces Lai et al.’s [122] findings that it often takes weeks to update trial insurance policies if the number of sites and estimated number of participants in a country changes.

COPCOV’s insurance procurement for two countries with differing levels of trial insurance experience, Nepal and Pakistan, point to both specific procedural challenges that sponsors face in instating trial insurance policies and broader issues of power in the global governance of trial insurance.

Newline insured one of the first COVID-19 trials in Nepal. Nepal did not have a national mandate for trial insurance and the University of Oxford previously provided blanket master policy coverage for its trials, such as the Oxford University Clinical Research Unit-Nepal (OUCRU) typhoid vaccine trial (Interviews 59–60). Following Oxford’s COVID-19 risk trigger, it took COPCOV investigators seven months to finalise a local insurance policy with a new local front. As an investigator in Nepal reflected, trial insurance is a relatively new concept in Nepal, which meant that, “we had to learn very quickly, you know, that all of this is required, and you have got to do this and, and even the insurer is unsure about what the heck we are insuring here, what’s going on. You know they’re all new in the game, and then there is also the usual bureaucracy…” (Interview 60). Instating insurance was further complicated by the fact that the intended trial site, OUCRU, did not have the legal status required by Oxford for initiating a policy (Interview 59, [65, 104]).

By the end of 2020, investigators at MORU highlighted that, “insurance is the only thing preventing recruitment from beginning in Nepal” and requested the University of Oxford to allow the trial to proceed while waiting for final payment of the policy, based on the argument that “the risk to delaying the study further is greater than the risk of not having insurance in place” [108]. However, university research services responded that it was “highly unlikely that anyone would confirm it would be OK to proceed without cover in place, as this leaves the university open to financial risks that it wishes to avoid” [67]. Research services suggested that the only options were to continue to wait to procure the policy through the University of Oxford, or, because they believed that “Nepal is not a particularly litigious nation (so the risk to the department should be rather limited in nature)”, MORU’s Tropical Medicine department could choose to “bear the risk of commencing the study prior to the insurance being in place” and provide proof of their financial ability to pay any claims [64]. MORU agreed to assuming liability in February 2021. Yet, because many healthcare workers had received the COVID-19 vaccine and COVID-19 infection incidence had by then decreased in Nepal, site initiation was halted and the trial was only activated when cases picked up again in July 2021 [103].

Pakistan has a longer history of clinical trial insurance than Nepal but setting up its insurance policy still took four months. In this case, the delays were less due to the capacity of the local front than payment issues and rigidity of sponsor requirements [71, 143]. MORU investigators requested a policy with Newline in mid-July 2020 [72, 176, 193]. Paperwork was convoluted between the clinical trials unit at the local site, local insurance front, and Newline [66, 109]. There were also challenges with convoluted payment systems required by the University of Oxford’s research and contract services [74, 105], including no option to expedite invoices [107, 113]. Ultimately, despite the fact that the local site, Aga Khan University, had previously worked with the local insurer, EFU Insurance [143], overseas payment delays and cash-before-cover requirements meant that the policy was not in force until November 2020 [68, 112, 107]. The trial site had limited opportunity to recruit before vaccines were rolled out to healthcare workers in January 2021 [106].

Interviews with other sites reveal concerns about the assurances provided by master and local policies. In Mali, investigators explained that they want a local policy because contact information for international insurers had often not worked in-country previously and because of banking issues (Interviews 38–40). In Indonesia, Laos, and Niger, investigators highlighted how the trial insurance policy might not synchronise with local or national hospitalisation payment systems if a participant had a severe adverse event. In this case, it might only be useful in catastrophic contexts. Jakarta site investigators explained that medical care is obtained through national health insurance at the local hospital, and investigators have never submitted claims for trial insurance in the past because it would preclude treatment at this site (at which patients are typically discharged after three days when they hit a national insurance payment limit, Interviews 15 and 18). The Medan site highlighted similar concerns with trial insurance, stating that they save it for extreme cases, as it would likely not pay out in time to cover any medications needed by participants (Interviews 22, 29 and 30). In Yogyakarta, the private hospital site lacked standard operating procedures for integrating trial participation into patient medical records, so it was unclear how claims would be triggered and paid (Interviews 16, 21). An investigator in Niger similarly described how:

I don’t think every trial needs insurance but I think if there’s a new drug or situation or something where there’s a CEO [profit incentives], there’s a genuine risk there. I think, you know, people really need to be protected… But what would we do if someone came to harm from one of the drugs? What I guess really worries me is whether if we ever had to use it, whether it would actually deliver, especially in a country like Niger where the health system is very weak in critical care and emergency care. I wouldn’t want to do a very high-risk study alone anyway, because I don’t think that infrastructure here supports it sufficiently. But if we did want to do a low-risk one, and we needed insurance, yeah, I guess in my heart of hearts I don’t believe that person would ever get it [compensation] (Interview 61).

Broadly, Oxford/MORU faced the challenge that local fronts “are not getting much out of it”. This meant that, “if we keep badgering, if we keep hassling the local supplier…they’re going to say, ‘we don’t need your business, you need us, we don’t need you’…that’s why they’ve started to charge a bit more now, this is because otherwise they’re just, they’re just providing paper for very little, little risk to them” (Interview 66). Translating trial insurance policy into practice is far from straightforward, and policies may sometimes be ticking a regulatory box and protecting sponsors from catastrophic liability rather than providing assurances of medical care for severe adverse events for local participants (Interview 18).

## Discussion

While some researchers have promoted trial insurance based on a belief that it “directly incentivizes sponsors of research to reduce risk” by linking premiums to institutional safety records [163], many are sceptical that insurance systems encourage academic researchers to self-limit trials with high medical risks (e.g., [164]). The current trial insurance system may in fact *over*-incentivise gatekeepers at academic institutions to mitigate potential reputational risks. Risk aversion behaviours generally increase with uncertainty (Interview 5, [99]), and risk management bureaucracies are most likely to respond to the threat of a low probability but high-cost event [134, 157] – like reputational fall-out from a major clinical trial during a pandemic – with obstructive mitigation precisely when clinical research to guide countermeasures is most needed. Mello et al. [136] refer to this impulse as a “natural tendency” for regulatory bodies to “become more conservative…pushed into a legalistic mood in which slavish attention to regulatory detail crowds out reviewers’ ability to ask real questions about the risks and benefits of research studies”.

Indeed, risk calculations underpinning the University of Oxford’s insurance requirements were made by research services and insurance company representatives (not principal investigators), and were not primarily focused on perceived medical risks of hydroxychloroquine [123] as an IMP or the trial's design [89]. They were not based on the inclusion of vulnerable populations in the trial (pregnant women had already been excluded based on the advice of the UK medical regulatory agency, [37, 44, 153]). They were also not based on past precedent or insurance premium costs. Neither MORU nor the University of Oxford had ever had a major claim over the past forty years. And, they were not motivated by ethics committee concerns,there is no evidence of any detailed analysis of insurance policy documents by ECs approving the COPCOV trial. As a research services gatekeeper at the University of Oxford explained, risk calculations were instead primarily based on the reputational risk of high-profile COVID-19 research being conducted outside of the United Kingdom:

It’s always been the case that it’s my reputation and it’s my job to protect the university… from my point of view, reputational damage is everything, especially in some of these territories that we go to consistently for a number of studies… so I’ve always taken a more risk averse side of it. It was never done laissez faire before but I think that it was a case of, “my dogs never run out of the road so we’re never going to shut the gate”. But you never do know about the stupid dog, that dog might run out. So I’d always shut the gate. It’s unlikely, yes, that something will happen. But just because it hadn’t happened in the past, doesn’t mean it’s not going to happen going forward. And that’s the whole point of insurance. It’s a risk transfer mechanism... I mean there was a stat someone told me the other day about how many hits [on the Oxford research website] we got during the pandemic… you could have thousands and thousands of positive headlines, but they only take one bad one and people focus on the bad one (Interview 66).

The COPCOV case study raises three reflections about risk frameworks at universities and the barriers posed by current trial insurance mechanisms. Firstly, gatekeepers have significant power in the interpretation of risk – and they often channel private sector financial management principles. Management consultants have explained how risk as a condition of self-aware individuals, such that organisations are incapable of being in direct risk. In this sense, universities may be best viewed as *conduits* through which individual gatekeepers – like risk managers in research services departments – take and transfer financial and reputational risk [96]. From an investment perspective, risk management has risen as a field of economics to amplify expected financial returns to individuals or organisations (yields), reduce anything that would make yields variable or uncertain (risk), and transfer risk to net profits (through insurance, [60, 130, 156]). This aligns with an insurance company’s recent description of clinical trial risk as “liabilities to third parties, reduction in the value of physical and intellectual property, interruption to activities, and reputational damage” [131].

In the 1960s, some sociologists surmised that such an “orientation toward profitable products and the resulting management demands in business firms and the professional ethos of university-trained scientists would clash permanently” [181]. Instead, risk managers and financialised definitions of risk have been deeply embraced by most university administrations. The University of Oxford’s multi-million-pound contract with an insurance broker and lawyers on retainer to negotiate liability with third parties like CROs [42] are standard practice [162].

Secondly, the current governance ecosystem for insurance makes it challenging for institutions with less financial and administrative resources to undertake multi-country trials and for members of some demographics to be included in trials. A Croatian professor foresaw this trend three decades ago, declaring at a WHO workshop on GCP harmonisation that, “as in all things, there are also negative aspects of GCP. The main one is the danger that, due to its heavy and costly bureaucratic endeavours, only those trials that gain industrial support will be performed” [200]. This claim has since been echoed globally (e.g., [196]). In the case of insurance, deciding whether to procure a policy depends on a combination of national requirements, scope of underwriting licenses by a master policy holder on retainer, and whether the sponsoring institution can absorb the costs of any claims if is un- or under-insured. Large for-profit companies have a higher ability to procure comprehensive master policies and to absorb financial risks than universities and smaller organisations, particularly those in LMICs, and are likely to negotiate better details for insurance premiums.

This system presents challenges for research with vulnerable populations during emergencies. For example, the United Kingdom’s multi-centre PROTECT-CH study sought to test prophylactic therapies in care homes during COVID-19. It experienced significant problems negotiating terms for care home trial insurance because this was a “product hitherto unavailable, at a time when insurers were extremely reticent about care home insurance in general” [82]. Hiring CROs to assist with recruitment and trial bureaucratic requirements can help in many contexts. However, CROs prefer larger contracts and are used to multi-million-pound operating budgets, which are often precluded for trials funded by research grants ([25, 115, 141, 152], Interview 5). If unchecked, cumbersome reinsurance processes may promote the concentration of clinical trials in a narrow number of countries within the Global South for which insurance and ICH-GCP compliance infrastructure is more developed [132]. Limited clinical trial pro-bono indemnification initiatives from insurance companies (e.g., [28] did little to fill gaps during COVID-19). Normative actors like the WHO provide little guidance to help academic research institutions in LMICs navigate insurance requirements,the details of the WHO’s no-fault global liability insurance mechanism for the Solidarity trial and COVAX were themselves largely untransparent [39, 41, 201, 202].

Thirdly, requirements for locally issued insurance documents do not and will not necessarily lead to enhanced protections for local populations or capacity development for clinical trials in LMICs. The national and local barriers experienced during COPCOV (Table 4) reflect how requirements for trial insurance policies are often at odds with the capacities of an insurance industry that is “either in its infancy, or very misunderstood, or just not something that’s available in that country” (Interview 66). These capacities are unlikely to evolve through the small risk and reward enshrined in reinsurance. The analysis of insurance policy documents from COPCOV’s Trial Master File revealed no details about how policies would integrate with national insurance policies or site hospital admission protocols in the case that a participant was harmed. There is also no data that sponsors’ insurance companies reliably pay claims initiated by injured participants [24, 95, 116]. There *is* data to show that insurance policy documents and terms are inconsistent and often technically challenging for ethics committees. Studies show confusion of ethics committees and regulatory bodies with insurance contractual language, particularly when it relates to the widening net of data monitoring committees [22, 59, 188], and a wide variety of language used in insurance policy documents (e.g., limits listed variously per claim, as total liabilities, as aggregate liabilities only, and with unclear deductibles and a variety of preconditions, [76]). As Ghooi and Divekar [76] reflected when analysing how ethics committees in India consider insurance policies, “…we were struck by the fact that compared to insurance, how simple science is”.

## Conclusion

All trial stakeholders interviewed indicated that they agreed in principle with some form of compensation mechanism acting as an ethical check-and-balance within the clinical research system. Yet, ballooning bureaucracies at academic institutions and contracted for-profit partners are largely divorced from on-the-ground questions at ethics bodies about whether insurance policies would pay out in a local context, what constitutes a fair pay-out, and their implications for chronically under-representing certain demographics from clinical research. Commercially provided clinical trial insurance is instead best viewed as part of a broader culture of mergers and acquisitions and risk outsourcing by academic institutions and pharmaceutical companies. These include CROs, site-management organisations (SMOs), shipping companies specialising in IMPs and samples, and insurance companies contracted to protect these shipments.

Insurance procedural barriers experienced during COPCOV (Table 4) can be productively viewed as part of this “python’s embrace”. Risk guardians at research institutions contribute to the python’s strength. Pournelle’s iron law of bureaucracy predicts that individuals devoted to the benefit of a bureaucracy ultimately gain control of it, while those dedicated to its goals (e.g., science production) are gradually diminished over time [97] . If risk managers in research services departments and clinical trial units are tasked with reputational protection, and if they use the expertise of brokers and a trail of insurers to contract out this risk, it is likely that bureaucratic structures – and documentary encumbrances – around financial risk exposure containment will continue to grow (Interview 5).

This presents a major question: is there another way to conceptualise risk for clinical trials and to pool risks that is less focused exclusively on harm mitigation [92] and more aligned with trial capacity building for academic institution-sponsored RCTs in LMICs? One pathway for clinical trial indemnification reform would take a “common-sense” approach. This would focus on addressing discrete bottlenecks within the existing insurance system. For instance, research services departments at universities could instate internal reforms to increase transparency of their insurance contracts and expedite their contractual approval and payment systems. More systematic availability of insurance requirements at the country level, such as through the WHO, and compilation of premium costs [46] could help smaller organisations looking to procure insurance to enter the competitive marketplace less blindly, without having to commission brokers and gain access to proprietary databases like Axco. Additionally, mentorship programmes could encourage countries to streamline insurance requirements. This could include (re)considering cash-before-cover and banking requirements and improving trial insurance policy terms’ alignment with existing national policies. Regional bodies could also establish systems supporting the alignment of insurance coverage through existing networks, such as AVAREF (Interviews 1–3).

Returning to the alternative models proposed for compensation (Table 2), how could Atuire’s “de-barriering” framework improve clinical trial capacity and (better) support the ethical principles of beneficence, justice, and respect for persons? De-barriering the clinical trial ecosystem will require high-level consultations on alternatives to sponsor-mediated insurance. For instance, the authors of an article on HIV/AIDS and National Institutes of Health trials in Africa suggested that a global fund could be created to fund research injury compensation. Such a fund could address inequalities in the treatment and compensation of participants and could be accompanied by the adoption of uniform regulations for no-fault compensation [128]. It would address the longstanding reality that smaller and less elite institutions have more trouble procuring commercial insurance with good terms.

This article does not provide a precise policy recommendation for this more systemic approach. Instead, I reinforce Atuire’s point that historical grounding and case studies are essential for identifying barriers to academic clinical trials, and that building coalitions of Global South actors is necessary to creatively re-think a system that is not currently fit for purpose. Major reform will, at a minimum, require improved transparency about international policy terms and costs, international guidance, and dedicated funding [26, 140]. As Kurihara et al. [120] highlights, there is a dire need for further surveys and international exchanges of information on countries’ compensation policies – including data on the incidence of injuries and status of compensation claims. Ongoing efforts to strengthen global clinical trial capacity, including the WHO’s Clinical Trials Resolution (WHA 75.8) and Good Clinical Trials Collective, should include deeper discussions about how to reform – or entirely rethink – trial indemnification.

## Supplementary Information

Additional file 1.

## Data Availability

The literature review and its coding are available on request in the form of an Excel document. The COPCOV stakeholder interview transcripts, emails, and Trial Master Files analysed during the are not publicly available, in compliance with GDPR and anonymisation as per the University of Oxford’s ethics clearance (CUREC) requirements. However, some of these data and all non-public files cited are available on reasonable request from the author, subject to the removal of any personally identifying information. A list of confidential emails and documents used to analyse the costs of the COPCOV trial’s insurance is provided in the supplementary file.

## Abbreviations

APBI: Association of the British Pharmaceutical Industry
AVAREF: African Vaccine Regulatory Forum
COPCOV: Evaluation of hydroxychloroquine or chloroquine for the prevention of COVID-19 (trial)
COVAX: COVID-19 Vaccines Global Access Facility
CRO: Contract research organisation
DNDi: Drugs for Neglected Diseases initiative
EVD: Ebola virus disease
EDCTP: European and Developing Countries Clinical Trials Partnership
GCP: Good clinical practice
HICs: High-income countries
ICH: International Council for Harmonisation of Technical Requirements for Pharmaceuticals for Human Use
IMP: Investigational medicinal product
IRB: Institutional research board
LMICs: Low- and middle-income countries
MORU: Mahidol Oxford Tropical Medicine Research Unit
NASEM: United States National Academies of Sciences, Engineering and Medicine
NIH: United States National Institutes of Health
OUCRU: Oxford University Clinical Research Unit (Nepal)
PRINCIPLE: Platform randomised trial of treatments in the community for epidemic and pandemic illnesses
PROTECT-CH: PROphylactic TrEatment of COVID-19 in Care Homes (trial)
PSI: Population Services International
RCT: Randomised-controlled trial
RECOVERY: Randomised evaluation of COVID-19 therapy (trial)
SMO: Site-management organisation
WHA: World Health Assembly
UHC: Universal health coverage

## Footnotes

1 For example, insurance terms can include liability, indemnification, compensation, insurance, or no-fault; the term clinical trial can be variously described as clinical research, RCTs, medical experimentation, or controlled trial; and global or regional trials are termed multi-centre, multi-country, trans-national, international, or regional. This scoping review is not registered but details of the protocol are available from the author on request.

2 MSF Access Campaign estimates that Phase II-III clinical trials range from $5 million - $142 million, and its TB-PRACTECAL clinical trial was the first to provide a detailed breakdown of all trial costs [6, 142]

3 European countries’ self-reported claims broadly indicated that most countries dealt with less than thirty claims total over the prior ten years – and that damages awarded were variable but insignificant compared to national budgets (with aggregate claims including administrative costs to insurance companies in the €200,000 to €550,000 range over ten years).

4 In this formula, C is the amount of compensation to be given to a participant, B is the base payout amount (INR 800,000), F is the age factor (ranging from 99.37 for ages 65+ to 228.54 for children under 16, based on workmen’s compensation), and R is the risk factor (representing the severity of disease, ranging from 0.5 for terminally ill participants to four for healthy volunteer participants). As of 2013, the government capped compensation at 73,600,000 INR and gave a minimum payout of 4,000,000 INR [33].

5 This total is based on 2021 average currency conversion to GBP and excludes Russia and Ukraine premiums as well as any reimbursement received from cancelled Myanmar and Vietnam policies
