# Supplementary material for: A python’s embrace? Insurance and the global clinical trial
Source: Health Res Policy Syst. 2026 Feb 9;24:23. doi: 10.1186/s12961-026-01449-6 (PMC12983545; doi:10.1186/s12961-026-01449-6)
Supplement: Supplementary file 1 — Additional file 1. [file 12961_2026_1449_MOESM1_ESM.docx]

**Supplement I: Search protocol for clinical trial insurance literature review**

The structured literature review of clinical trial insurance practices, coverage options, and critical perspectives on clinical trial-specific indemnification mechanisms was conducted in January 2025 in PubMed and Europe PMC. Search terms included:

1. “clinical trial*” AND insurance AND “randomised controlled trial” OR “randomized controlled trial” OR “RCT”
2. “clinical trial*” AND liability; liability AND pandemic AND trial*
3. “no-fault” OR “no fault” AND trial* AND insurance
4. compensation and “clinical trial*”

Searches were limited to titles/abstracts, articles published before 1 January 2025, and articles with a title or abstract translated into English. A total of 2,630 articles were identified through these searches, of which 127 were included in the literature review. Articles were included if they focused on clinical trial-specific insurance, indemnity, or compensation for injury *and* provided details about policy options or experiences with insurance in the context of a trial. Articles were excluded from analysis if they focused on health insurance claims databases, trial participant costs for cancer studies/financial toxicity/healthcare reimbursement (a separate issue from compensation due to unforeseen injury), use of health insurance plans for trial recruitment, broader medical insurance company statistics, and cost-benefit analyses for insurance company’s use of specific therapeutics. The review does not include specific searches for country-level laws and guidance for clinical trial insurance, as this would require a major separate analysis and language skills (e.g., using databases like Westlaw in the United States [Obeng-Gyasi et al. 2019] and systematically searching each country’s government websites). The review is of a historic nature and not systematic, and it was conducted by a single author. Future more targeted searches (restricted by trial insurance topic, geography, and years) could be more reproducible and more systematically include literature from non-medical fields (e.g., using Scopus and ProQuest to search for references to trial insurance in social science literature).

The scoping literature review was supplemented by Google searches for grey literature, industry publications, and reflections on clinical trial insurance practices, using the search term “clinical trial insurance”. It was further supplemented by primary sources about GCP and trial insurance from the World Health Organization Archives (Geneva), GSK Archives (London), and Novartis Archives (Basel). It did not include specific searches for country-level laws and guidance for clinical trial insurance, as this would require a much wider search scope and language skills (e.g., using databases like Westlaw in the U.S.^[[1]](#footnote-1)^ and systematically searching each country’s government websites). Results were summarised using a matrix approach in Excel and analysed chronologically.

**Supplement II: Sources for COPCOV costs and insurance policy details**

Sources were shared confidentially with the author by COPCOV trial investigators. These included more than 3,000 emails and insurance documents from the Trial Master File. Details of emails cited in the text and retained by the author include:

1. Gary Priest to Heather House, Nick Day, Nick White, William Schilling, Salwaluk Panapipat. 23 March 2020 17:11. FW: COPCOV – Asia arm.
2. Gary Priest to Heather House, Nick day, Nick White, William Schilling, Salwaluk Panapipat, 23 March 2020 09:59. COPCOV – Asia arm.
3. Gary Priest to James Callery, Heather House, John Minogue. 28 January 2021 21:30. RE: COPCOV insurance review: Jan 6 update.
4. Gary Priest to Nick White, William Schilling, Salwaluk Panapipat. 16 March 2020 20:37. RE: COPCOV COVID19 trial.
5. Gary Priest to OxTREC Ethics, Tanya Cope, Heather House. 15 April 2020 4:10 PM. RE: COPCOV.
6. Gary Priest to Tanya Cope. 4 June 2020 12:26 AM. RE: Oxford/MORU and Biorasi: insurance arrangement.
7. Gary Priest to Tanya Cope, John Minogue, James Callery, William Schilling. 18 November 2020 11:27. RE: COPCOV insurance review: Nov 16 update & next steps.
8. Gary Priest to Tanya Cope, John Minogue, James Callery, William Schilling. 18 November 2020 17:17. RE: COPCOV insurance review: Nov 18 update & next steps.
9. Gary Priest to Tanya Cope. 10 February 2021 8:23 PM. RE: COPCOV insurance review: Feb 1 status.
10. John Minogue to Gary Priest, James Callery. 27 October 2020, 14:28. Insurance invoices – paid.
11. Nick White to Gary Priest, William Schilling, Salwaluk Panapipat. 16 March 2020 13:32. RE: COPCOV COVID19 trial.
12. Saeed Hamid to James Callery, Tanya Cope. 30 September 2020 10:31. RE: COPCOV Pakistan.
13. Tanya Cope to Gary Priest. 3 June 2020 15:28. FW: Oxford/MORU and Biorasi: insurance arrangement.
14. Tanya Cope to Gary Priest, “Re: COPCOV insurance review: Apr 5 status”, 14 April 2021 13:59.
15. Tanya Cope to Gary Priest, “RE: COPCOV insurance review: Mar 12 status”, 12 March 2021 04:58.
16. Tanya Cope to Gary Priest. 24 July 2020 07:01. RE: COPCOV: insurance summary w/ V5: status check & new African requests.
17. Tanya Cope to Gary Priest. 4 June 2020 07:11. RE: Oxford/MORU and Biorasi: insurance arrangement.
18. Tanya Cope to Gary Priest, Rebecca Bryant. 15 July 2020 11:21. RE: COPCOV: insurance summary w/ V5.
19. Tanya Cope to Gary Priest, Rebecca Bryant. 4 July 2020 2:40 PM. RE: COPCOV: insurance summary w/ V5.
20. Tanya Cope to James Callery, William Schilling, Gary Priest, John Minogue. 5 November 2020 04:40. RE: COPCOV insurance review: notes & actions from Monday call.
21. Yan Sukhanov to Alexander Romanov, David Burton, William Schilling, Nick White, Nick Day, Tanya Cope. 3 June 2020 8:33 PM. RE: Oxford/MORU and Biorasi.
22. Activa assurances, certificate of insurances, Policy no. 12001-09005/1001/180 0000 621, 12 November 2020.
23. Activa assurances, contrat d'assurance, responsabilite civile essai clinique (clinical trials liability), no. police: 12001-09005/1001/180 0000 621.
24. Assurance responsabilite civile objective experimentations, conditions particulieres, police no. 2020-10-8218288, 17 December 2020.
25. Asuransi MAG, Endorsement.
26. Asuransi MAG, Policy of insurance, Policy no. 45090120000363.
27. Bangkok Insurance (Lao) Company Limited, Premium invoice, No. 1,046, 11 April 2020.
28. Certificate of insurance, Policy no. 230/100007886, L'Africaine des Assurances, 14 December 2020.
29. Certificate of insurance, Policy no. P/01/2010/332097/2020, 20 July 2020.
30. Green Delta Insurance, Certificate of Insurance for Clinical Trials, Policy GDI/PBD/02/2021/PLI/0006, issued 7 February 2021.
31. H.W. Wood Limited, To whom it may concern, Re: Medecins Sans Frontières International, 10 November 2020, Policy no. L200792. EGU General Insurance ltd, Online payment 315, dated 05 Nov 2020.
32. Jubilee Insurance, Certificate of insurance, Policy no. P/101/6016/2020/000007, 30 July 2020.
33. Jubilee Insurance, Clinical trial liability insurance policy schedule, retroactive date 6 November 2019.
34. Madison General, Risk details, 20 July 2020.
35. Nallias - SA, No fault compensation insurance for clinical trials and/or human volunteers studies, Schedule, Policy number 6010003, 14 May 2021.
36. NecoInsurance Ltd., Debit Note, 9 June 2021, M/s B.P. Koirala Institute of Health Sciences.
37. Newline Group, Newline Syndicate 1218 at Lloyd’s & Lloyd’s Insurance Company SSA, Clinical Trials Insurance Quotation for: University of Oxford, Quoted 29 June 2020, Proposed Programme of Coverages, Clinical Trial Protocol No: COPCOV.
38. Newline Group, Newline Syndicate 1218 at Lloyd’s & Lloyd’s Insurance Company SSA, Clinical Trials Insurance Quotation for: University of Oxford, Quoted 11 November 2020, Proposed Programme of Coverages, Clinical Trial Protocol No: COPCOV.
39. NIA, Attestation d'Assurance, No. 00608862, 24 September 2020.
40. Nib Insurance Company, No fault compensation insurance for clinical trials and/or human volunteers studies, Policy no. P/009/27/04/2021/01/00001, 9 January 2021.
41. Police d'Assurance responsabilite civile, Police 618 862, 20 November 2020.
42. Police n. 230/100007886, Assurance responsabilite civile essais cliniques, Universite de Oxford.
43. Russian Federation. 2011. Government Decree No. 714 of September 13, 2010, on approval of typical rules for compulsory insurance of the life and health of a patient involved in clinical trials of a medical product (as amended by Government Decree of May 18, 2011, No.393).
44. Yan Sukhanov to Alexander Romanov, David Burton, William Schilling, Nick White, Nick Day, Tanya Cope. 3 June 2020 20:32. RE: Oxford/MORU and Biorasi.

**Supplement III: Stakeholders interviewed**

Trial stakeholders were identified in consultation with the principal investigators. A list of all site investigator teams was compiled for both prospective and recruiting sites, and JW systematically contacted all stakeholders requesting an interview (and snowballing for additional local investigators). Interviews were conducted on Microsoft Teams and, in some cases, in-person in the United Kingdom, Indonesia, and Thailand. Background about the researcher (JW) was provided to all participants through a “one pager” about the project and its themes and an extensive participant information sheet. Semi-structured interviews were based on a short interview guide with approximately ten general questions about the participants’ clinical trial background and experiences during COPCOV, including narrative questions about the participant’s background and case study questions focused on barriers and facilitators of trial approval, site activation, recruitment, supplies/drug and sample shipping (as relevant), and site closure/data analysis (as relevant). When permission was given to record audio, interviews were transcribed and stored according to the CUREC approval requirements at the University of Oxford (on OneDrive), and were analysed by JW, as per CUREC requirements.

| **Interview number** | **Stakeholder** | **Interview date(s)** |
| --- | --- | --- |
| 1 | AVAREF secretariat stakeholder | 6 September 2022, 10 November 2022 |
| 2 | AVAREF secretariat stakeholder | 6 September 2022 |
| 3 | AVAREF secretariat stakeholder | 6 September 2022, 25 October 2022, November 2022 |
| 4 | COPCOV co-principal investigator | Monthly sessions February 2022-August 2024 |
| 5 | COPCOV co-principal investigator | 26 April 2022, 10 May 2022, 25 August 2023, 5 September 2023 |
| 6 | COPCOV data safety management committee member | 10 June 2023, 13 June 2023 |
| 7 | COPCOV trial steering committee member | 20 May 2022 |
| 8 | Ethiopia site investigator | 7 March 2023 |
| 9 | Former Epicentre clinical trial researcher | 24 May 2024 |
| 10 | Former scientific director DNDi | 1 November 2022 |
| 11 | Ghana prospective site investigator | 16 June 2023 |
| 12 | Good manufacturing practice (GMP) specialist | 21 November 2022 |
| 13 | Guatemala prospective site investigator | 27 February 2023 |
| 14 | Indonesia site administrator | 30 August 2023 |
| 15 | Indonesia site chief investigator | 1 September 2023, 2 September 2023 |
| 16 | Indonesia site coordinator | 29 August 2023 |
| 17 | Indonesia site coordinator | 7 September 2023 |
| 18 | Indonesia site coordinator and trial monitor | 27 April 2023, 1 September 2023, 2 September 2023 |
| 19 | Indonesia site data manager | 7 September 2023 |
| 20 | Indonesia site data manager | 7 September 2023 |
| 21 | Indonesia site investigator | 29 August 2023 |
| 22 | Indonesia site investigator | 4 September 2023 |
| 23 | Indonesia site laboratory manager | 30 August 2023 |
| 24 | Indonesia site lead investigator | 15 February 2023 |
| 25 | Indonesia site nurse | 30 August 2023 |
| 26 | Indonesia site nurse | 7 September 2023 |
| 27 | Indonesia site pharmacist | 30 August 2023 |
| 28 | Indonesia site pharmacist | 7 September 2023 |
| 29 | Indonesia site study doctor | 4 September 2023 |
| 30 | Indonesia site study doctor | 4 September 2023 |
| 31 | Indonesia site study doctor | 30 August 2023 |
| 32 | Indonesia site study doctor | 30 August 2023 |
| 33 | Indonesia site study doctor | 7 September 2023 |
| 34 | Indonesia site study nurse | 30 August 2023 |
| 35 | Insurance broker representative | 10 September 2023 |
| 36 | Insurance company representative | 26 August 2023 |
| 37 | Italy prospective site investigator | 13 April 2023 |
| 38 | Mali site administrator | 3 May 2023 |
| 39 | Mali site coordinator | 3 May 2023 |
| 40 | Mali site lead investigator | 3 May 2023 |
| 41 | Medecins Sans Frontieres clinical trial expert | 4 December 2024 |
| 42 | MORU, administrator | 14 June 2022 |
| 42 | MORU, chief operating officer | 23 June 2022, 24 August 2023 |
| 43 | MORU, clinical trial support group representative | 27 May 2022 |
| 44 | MORU, clinical trial support group representative | 5 June 2022 |
| 45 | MORU, clinical trials support group representative | July 2022, August 2023 |
| 46 | MORU, clinical trials support group representative | April 2022, May 2022 |
| 47 | MORU, COPCOV monitoring lead | 22 June 2022 |
| 48 | MORU, COPCOV site coordinator | 29 April 2022, 23 June 2022, 25 August 2022, 8 September 2023 |
| 49 | MORU, COPCOV study coordinator | 24 August 2022, 26 September 2022, 10 March 2023 |
| 50 | MORU, data manager | 15 June 2022, 23 June 2022, 14 September 2023 |
| 51 | MORU, director | 23 June 2022 |
| 52 | MORU, financial administrator | 21 June 2022 |
| 53 | MORU, financial coordinator | 21 June 2022 |
| 54 | MORU, financial manager | 5 June 2022 |
| 55 | MORU, researcher | 15 June 2022 |
| 56 | MORU, shipping and logistics expert | 8 February 2023 |
| 57 | MORU, Thailand site coordinator | 22 June 2022 |
| 58 | MORU, trial statistician | 28 May 2022 |
| 59 | Nepal site coordinator | 24 August 2023 |
| 60 | Nepal site lead investigator | 9 March 2023 |
| 61 | Niger site investigator | 21 February 2023 |
| 62 | Pakistan site lead investigator | 17 April 2023 |
| 63 | Sanofi pharmaceutical access specialist | 6 December 2024 |
| 64 | Sanofi pharmaceutical risk specialist | 31 January 2024 |
| 65 | United Kingdom country lead investigator | 1 June 2022 |
| 66 | University of Oxford insurance specialist | 29 March 2023 |
| 67 | Vietnam prospective site investigator | 22 February 2023 |
| 68 | WHO Guideline Review Committee representative | 9 May 2023 |
| 69 | Zambia site investigator | 2 March 2023 |

1. Obeng-Gyasi et al. 2019. [↑](#footnote-ref-1)
